# New genetic findings lead the way to a better understanding of fundamental mechanisms of drug hypersensitivity

**DOI:** 10.1016/j.jaci.2015.06.022

**Published:** 2015-08

**Authors:** Munir Pirmohamed, David A. Ostrov, B. Kevin Park

**Affiliations:** a Department of Molecular and Clinical Pharmacology, MRC Centre for Drug Safety Science, University of Liverpool, Liverpool, United Kingdom; b Department of Pathology, Immunology and Laboratory Medicine, University of Florida College of Medicine, University of Florida, Gainesville, Fla

**Keywords:** Drug hypersensitivity, HLA, genetic polymorphisms, mechanisms, crystallography, ADR, Adverse drug reaction, p-i, Pharmacologic interaction, SJS, Stevens-Johnson syndrome, TEN, Toxic epidermal necrolysis

## Abstract

Drug hypersensitivity reactions are an important clinical problem for both health care and industry. Recent advances in genetics have identified a number of HLA alleles associated with a range of these adverse reactions predominantly affecting the skin but also other organs, such as the liver. The associations between abacavir hypersensitivity and HLA-B*57:01 and carbamazepine-induced Stevens-Johnson syndrome and HLA-B*15:02 have been implemented in clinical practice. There are many different mechanisms proposed in the pathogenesis of drug hypersensitivity reactions, including the hapten hypothesis, direct binding to T-cell receptors (the pharmacologic interaction hypothesis), and peptide-binding displacement. A problem with all the hypotheses is that they are largely based on *in vitro* findings, with little direct *in vivo* evidence. Although most studies have focused on individual mechanisms, it is perhaps more important to consider them all as being complementary, potentially occurring at the same time with the same drug in the same patient. This might at least partly account for the heterogeneity of the immune response seen in different patients. There is a need to develop novel methodologies to evaluate how the *in vitro* mechanisms relate to the *in vivo* situation and how the highly consistent genetic findings with different HLA alleles can be more consistently used for both prediction and prevention of these serious adverse reactions.

Adverse drug reactions (ADRs) remain a major problem in the clinic and in drug development.^1^ Of particular concern are unpredictable ADRs, which are often referred to as type B or idiosyncratic reactions.^2^ They can affect any organ system, most commonly the skin but also the liver, lungs, bone marrow, and kidneys. Such reactions are still poorly understood, but many of them are often assumed to be immunologic in nature,^3^ so-called drug hypersensitivity reactions.

Recent advances in the pharmacogenetics of drug hypersensitivity reactions, most notably HLA restriction, have provided persuasive evidence that some of these drug reactions have an immunologic component. Furthermore, such advances are beginning to provide the experimental tools to investigate the interplay between the genetic, cellular, and chemical mechanisms postulated to be involved in drug hypersensitivity. We now need to build on such advances in a critical and constructive fashion to:

• develop diagnostic tests to define whether an ADR to a new drug is truly immunologic;
• develop pharmacogenetics tests (which might be point of care) for patients to prevent these reactions, which would fit in with the evolving paradigm of personalized or precision medicine; and
• provide the science to develop novel *in vitro* model systems with which to (1) eliminate immunologic liabilities from drugs known to be associated with hypersensitivity and (2) develop preclinical test systems to minimize the immunologic liabilities of new drugs in development.

To achieve these goals, we need to investigate further the best understood case histories “from man to molecule and back again.”

## Genetic basis of drug hypersensitivity

### Association with HLA alleles

**Fig 1:**
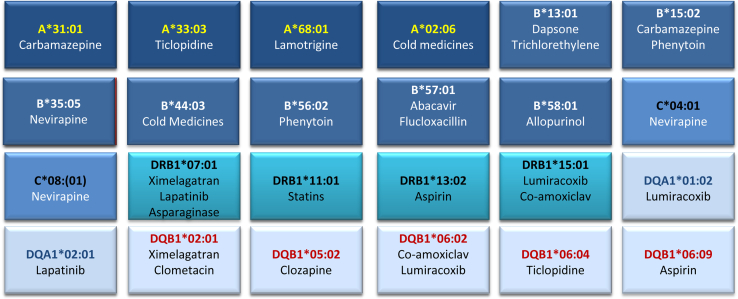
HLA associations reported with serious ADRs caused by many different drugs. This is not an exhaustive list of reported drug-HLA associations but illustrates that the pattern of drug–HLA–tissue injury interactions varies and that there is no general rule for predicting susceptibility.

Since the completion of the human genome project, there have been some remarkable advances in elucidating the genetic basis of drug hypersensitivity reactions.^4,5^ This has largely focused on the HLA alleles in the MHC on the short arm of chromosome 6. This is the region of the genome that is highly polymorphic and has been linked to both autoimmune diseases and infectious diseases. Since 2001, at least 24 ADRs have been linked to different HLA alleles (Fig 1). These associations afford a fascinating insight into the complexity of the immune response, highlighting our incomplete understanding of the pathogenesis of these reactions. However, they also provide several important insights that need to be addressed in future research.

First, different organ systems are affected by these ADRs, most commonly the skin and liver, but also muscles (statin-induced autoimmune myopathy and HLA-DRB1*11:01)^6^ and neutrophils (clozapine-induced agranulocytosis and *HLA-DQB1*).^7^ Whether the HLA allele determines which organ system is affected is unclear, but it is likely to play a role in association with other factors, such as other genetic variants, expression of organ-homing receptors, and T-cell clonotypes.

Second, the associations sometimes show marked ethnic variation reflecting the background prevalence of the implicated HLA allele. The most impressive example of this is the association of HLA-B*15:02 with carbamazepine-induced Stevens-Johnson syndrome (SJS) and toxic epidermal necrolysis (TEN) in Han Chinese, Thai, and Malay patients^8^ but not in Northern Europeans.^9^ The background prevalence of HLA-B*15:02 varies from 4% to 15% in the affected populations but is less than 1% in Japanese and Korean subjects and extremely rare in Northern Europeans (<0.01%).

Third, the same HLA allele can be associated with adverse reactions to therapeutically and structurally unrelated compounds but with effects in different organs. The best example of this is the association of HLA-B*57:01 with both abacavir hypersensitivity^10^ and flucloxacillin-induced hepatotoxicity.^11^ This is considered in more detail below.

Fourth, the same type of organ injury can occur with the same HLA allele, even with therapeutically and structurally unrelated compounds. For example, HLA-DRB1*15:01 is associated with liver injury with both lumiracoxib^12^ and co-amoxiclav.^13^ Whether there are some HLA alleles that predispose to certain forms of organ injury will become clear as more research is undertaken with other drugs but seems likely based on the possibility that certain HLA alleles can be more highly expressed in particular organs. Consistent with this, a recent genome-wide association study showed that HLA-DRB1*15:01 is also associated with alcohol-induced liver cirrhosis.^14^

Fifth, although most of the associations illustrated in Fig 1 are consistent with the time lag needed to induce an immune response (and are consistent with clinical and histologic evidence), for some forms of organ injury, the HLA association was surprising and highlighted that the general rules of immune-mediated injury occurring soon after the start of the drug or being worse on rechallenge are not necessarily correct in all cases. For example, lumiracoxib-induced liver injury typically occurs after more than 100 days on the drug^12^ which is not the usual timeframe associated with liver injury caused by other drugs, such as co-amoxiclav. For clozapine agranulocytosis, rechallenge does not necessarily result in recurrence of the reaction much sooner,^15^ which is inconsistent with evidence from other forms of immune-mediated drug injury, such as abacavir hypersensitivity.^16^

Finally, it is interesting to note that the drugs most commonly associated with cutaneous ADRs, such as the penicillins and sulfonamides, have not convincingly been shown to have associations with HLA alleles. This might be because they form multiple epitopes and therefore interact with multiple HLA alleles, but this cannot be the full explanation given the very strong association between flucloxacillin hepatotoxicity and HLA-B*57:01. A possible explanation is that studies thus far have not phenotyped the patients precisely with mixing of phenotypes, poor assessment of causality, and inadequate sample sizes. This is perhaps illustrated by a recent genome-wide study in which careful phenotyping of an adequate number of patients with penicillin-induced type 1 IgE-mediated reactions showed an association within the *HLA-DRA* region.^17^

### Association with non-HLA alleles

Observational studies have shown with some drugs, such as phenytoin, that higher doses, particularly at commencement, can increase the risk of cutaneous eruptions.^18^ Similarly, with lamotrigine, higher doses, particularly when the patient is cotreated with sodium valproate, which inhibits the glucuronidation of lamotrigine,^19^ lead to a higher frequency of serious cutaneous ADRs, including SJS.^20^ This led to revised prescribing information to start patients on lower doses of lamotrigine and escalate the dose slowly.

These clinical observations would suggest that genetic polymorphisms in drug metabolic pathways can increase the risk of serious idiosyncratic reactions. However, candidate gene studies and, more recently, genome-wide association studies have not shown any convincing associations with polymorphisms in genes coding for drug-metabolizing enzymes or transporters.^21^ The exception to this seems to be phenytoin; a recent genome-wide association study^22^ in Taiwanese, Japanese, and Malaysian patients showed a strong association with CYP2C9*3, an allelic variant associated with reduction in CYP2C9 activity by about 90%, which reached genome-wide significance (*P* = 10^−17^). There are perhaps several lessons to be learned from this.

First, phenytoin has a narrow therapeutic index because its fractional clearance is largely dependent on CYP2C9. Thus the lower activity of the CYP2C9*3 allelic variant leads to reduced metabolic clearance^22^ and higher plasma concentrations, which are consistent with clinical observations of a dose-dependent relationship in the risk of cutaneous eruptions.^18^ It also suggests that a relative deficiency in one pathway might not necessarily predispose to a serious ADR if multiple metabolic pathways are involved in the clearance of that drug. Alternatively, the effect size may be so low that very large numbers of patients would be required to show genome-wide significance.

Second, type B or idiosyncratic reactions have typically been classified as being dose independent.^2^ However, these more recent findings suggest that even these reactions have some form of dose-response relationship, but this might not be dependent on the external dose but more on the systemic exposure to the parent drug or 1 or more of its metabolites.

Third, this association with reduced metabolic clearance of phenytoin does not necessarily mean that the parent drug is responsible for the reaction because inability to metabolize phenytoin through its main route can lead to diversion through more toxic pathways.

### Clinical translation

The effect size and therefore the strength of association with HLA alleles varies with different drugs,^4,5^ which has influenced translation into clinical practice. To date, for abacavir hypersensitivity and carbamazepine-induced SJS in Southeast Asians, the drug label has been changed, stating that HLA testing needs to be done before commencing the drug.^21^

### Abacavir hypersensitivity

Abacavir hypersensitivity occurs in approximately 5% to 7% of patients.^23^ The reaction is CD8^+^ T cell mediated.^24^ The association between the HLA class I allele HLA-B*57:01 and abacavir hypersensitivity was first reported from Australia^25^ and North America^26^ and subsequently by a study from the United Kingdom.^27^ Genetic testing has been demonstrated to be cost-effective, first in the United Kingdom^27^ and subsequently in other countries.^28-30^ Furthermore, preprescription testing for HLA-B*57:01 reduced the frequency of hypersensitivity in Australia,^31^ the United Kingdom,^32^ and France,^33^ showing that genetic testing can have a powerful influence in reducing the burden associated with serious ADRs. This is covered in more detail in the article by White et al^34^ in this issue of the *Journal*.

### Carbamazepine-induced hypersensitivity reactions

Carbamazepine is widely used in the treatment of epilepsy, trigeminal neuralgia, and bipolar disorder. The clinical manifestations of carbamazepine hypersensitivity are more complex and diverse than those observed with abacavir and include simple maculopapular exanthema, hypersensitivity syndrome, or drug reaction with eosinophilia and systemic symptoms syndrome and SJS/TEN. Carbamazepine-induced SJS/TEN has shown a strong (odds ratio >1000) association with HLA-B*15:02 in the Han Chinese population.^35-37^ This association has also been replicated in several other Asian populations, including Thai,^38,39^ Malay,^40^ and Indian subjects^41^ but not in white^9,42,43^ and Japanese subjects.^44,45^ Apart from being ethnicity specific, the association with HLA-B*15:02 is phenotype specific in that it is only valid for SJS/TEN but has not been shown to be important for maculopapular exanthema and hypersensitivity syndrome.^36^ The utility of HLA-B*15:02 testing for preventing carbamazepine-induced SJS/TEN was also shown in a prospective study from Taiwan.^46^ The drug label for carbamazepine has been changed by many drug regulatory agencies worldwide, including the European Medicines Agency and US Food and Drug Administration. Even though genetic testing for HLA-B*15:02 is reimbursed in Hong Kong, advice that patients should be tested for this allele before the use of carbamazepine led physicians to use alternative anticonvulsants, such as phenytoin and lamotrigine, which did not reduce the overall incidence of SJS-TEN in Hong Kong.^47^ This provides a salutary lesson as to the difficulties that can be encountered in implementing genetic tests into clinical practice.

For other serious cutaneous ADRs caused by carbamazepine in Chinese patients^48^ and for maculopapular eruptions, drug reaction with eosinophilia and systemic symptoms syndrome, and SJS/TEN in white^49^ and Japanese^50^ subjects, HLA-A*31:01 has been shown to be a predisposing factor. A recent study has demonstrated that preprescription genotyping for HLA-A*31:01 in the context of the United Kingdom National Health Service would be cost effective.^51^ HLA-A*31:01 is mentioned in drug labels in many countries, but testing is not mandatory, and thus it is not routinely used in clinical practice. A recent functional study in a carbamazepine-hypersensitive patient showed the activation of both HLA-A*31:01–restricted, carbamazepine-specific CD8^+^ T cells and HLA-DRB1*04:04–restricted, carbamazepine-specific CD4^+^ T cells, indicating that cooperation between different T-cell subsets within the extended genetic haplotype plays a role in the clinical manifestations seen in patients.^52^

## Pathogenesis of drug hypersensitivity reactions

**Fig 2:**
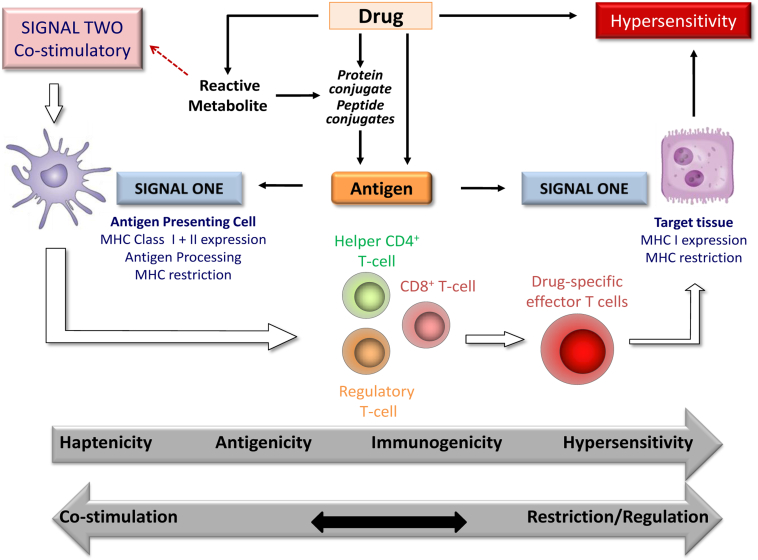
A scheme to illustrate current hypotheses concerning how a drug might interject in natural immunity to cause a hypersensitivity ADR. It is likely that multiple molecular initiating events, including reactive metabolite formation, conjugation of drug to protein, and direct interaction of the drug and MHC molecules, can provide signal 1. For a response to proceed, there will be regulation by costimulatory (signal 2) and coinhibitory molecules, which might ultimately activate various adverse pathways, resulting in the highly variable clinical manifestations of drug hypersensitivity. It is important to note that no mechanism has been proven unequivocally in human subjects to date. Furthermore, little is known about the signal in the target cell or tissue that provokes effector T cells to cause tissue damage.

Our current understanding of drug hypersensitivity is outlined in Fig 2. From a chemical perspective, the drug can act as:

• a *hapten*—a chemical that forms covalent attachment to a protein;
• a *prohapten*—a chemical that can be converted to a hapten;
• an *antigen*—a chemical, normally a peptide, that acts as a ligand between MHC molecules and cognate immunologic receptors in both the afferent and efferent limbs of the immune response;
• a *costimulatory agent*—a chemical or patient factor that interacts with dendritic cells, polarizing and maturing an immune response;
• an *immunogen*—a chemical that can initiate an immune response (in human subjects); and
• a *sensitogen*—a chemical that can elicit hypersensitivity (in human subjects).

From an immunologic perspective, it is important to note that the whole process is likely to be subject to immune modulation by costimulatory signals, regulatory T cells, and the nature of the antigen in the target tissue, which pulls the destructive immune response into the target tissue.

### The chemical basis of drug hypersensitivity

Until recently, the hapten hypothesis was the most widely assumed mechanism for drug hypersensitivity. The ability of low-molecular-weight nitrophenyl derivatives to cause skin sensitization in guinea pigs is a function of their chemical reactivity^53^: trinitrophenyl-modified MHC peptides can generate a T-cell response, and trinitrophenyl-specific T cells can recognize both trinitrophenyl-modified and unmodified peptides. This shows how chemical modification can break tolerance. Furthermore, such skin sensitizers indirectly activate innate inflammatory signaling through production of reactive oxygen species and hyaluronic acid degradation.^54^ These findings in experimental animals have been translated into human experimental models through topical application of dinitrochlorobenzene to volunteers,^55-57^ in whom both chemical-specific CD4^+^ and CD8^+^ T cells have been detected, which is consistent with the ability of 2,4-dinitrochlorobenzene to form haptens with both intracellular and extracellular proteins.

Penicillins are β-lactam antibiotics that react spontaneously with nucleophilic lysine residues in proteins in a dose- and time-dependent fashion.^58,59^ Antibodies have been detected against penicillin-protein conjugates and the benzylpenicilloyl group but not the parent drug.^60-63^ Use of specific mass spectrometric methods has shown that penicillin-protein conjugates can be detected in all patients exposed to the drug, and T cells that recognize protein conjugates^64,65^ have been demonstrated in hypersensitive patients. Interestingly, specific lysine residues are targeted in the albumin molecule at low concentrations, with the number of epitopes increasing as the drug concentration increases.^66^ In patients with cystic fibrosis, which is characterized by a high incidence of piperacillin hypersensitivity, mass spectrometric techniques have revealed the presence of multiple haptens on albumin in patients with the presence of drug-specific T-lymphocyte responses.^59^ Given that antibiotic courses are often given over 14 days in these patients and that the half-life of albumin is 19 days, modified protein is likely to accumulate during the course and will persist after the course. How this relates to the likelihood of hypersensitivity occurring during the course of the treatment or sometimes after the course is completed requires further investigation.

A key unanswered question for penicillins and the hapten hypothesis is why one penicillin, such as piperacillin, targets the skin primarily, whereas another, such as flucloxacillin or co-amoxiclav, can also target the liver. Organ-selective disposition of the drug antigen could be a factor (ie, albumin is synthesized in the liver),^67^ but it is also possible that organ-selective costimulatory signals could play a role given the ubiquitous nature of penicillin hapten formation. In relation to flucloxacillin, it is metabolized by CYP3A4 to 5′-hydroxymethylflucloxacillin,^68^ but the overall ratio of parent drug to metabolite is 25:1, and therefore whether this is important in hepatic toxicity is unclear.

The human safety risk posed by the generation of reactive metabolites with the potential to form haptens *in vivo* remains a major concern for drug development.^69^ A large number of drugs associated with hypersensitivity have been shown to form reactive metabolites that can modify proteins both *in vitro* and *in vivo*.^3^ However, the “reactive metabolite theory of drug hypersensitivity” is still largely based primarily on a “global association” between *in vitro* bioactivation and clinical outcome. General anesthetics were one of the first paradigms used to investigate the role of metabolism in drug-induced immune hepatitis. Halothane is metabolized in the liver by CYP2E1 to yield highly reactive trifluoroacetyl chloride, which binds spontaneously to proteins. Circulating IgG antibodies that recognize modified liver proteins and autologous proteins were implicated in the mechanism of halothane hepatitis because they were detected in all patients with severe reactions.^70,71^ Interestingly, the overall extent of metabolism (bioactivation) of enflurane and isoflurane, which have replaced halothane in clinical practice, is around 10-fold lower, and these are associated with a much lower risk of hepatitis.^2^ Similarly, IgG antibodies have been detected that recognize drug-modified proteins in patients who had immune-mediated hepatitis on isoniazid.^72^ More recently, a number of drugs known to cause liver injury have been shown to have strong genetic associations with specific HLA alleles (Fig 1). All these drugs are known to undergo P450-mediated metabolism, but whether the parent drug, stable metabolite, or chemically reactive metabolites are involved is undetermined at present. For example, with lapatinib, bioactivation to a quinoneimine intermediate has been shown,^73^ but again, the final proof of its role in hepatic injury is not available. The main issue is that at present, the technology to investigate T-cell responses to short-lived chemically reactive intermediates does not exist.

The ultimate proof of the hapten hypothesis would be detection and characterization of effector drug–peptide conjugates in a T-cell assay that simultaneously discriminates between hypersensitive and nonhypersensitive patients. The first step toward such an assay system has been the development of novel sensitive mass spectrometric methods that enable the full characterization and quantification of drug-modified proteins in patients. Such methods can be used not only to detect which proteins have been modified but also the precise chemistry of the hapten and the specific amino acid modified within the target protein. Such platforms provide a direct and unequivocal definition of the relationship between drug metabolism and hapten formation *in vivo* and provide sequence information for the synthesis and immunologic evaluation of potential antigenic drug-peptide conjugates.^59^

In summary, the above examples provide evidence that bioactivation (either spontaneous or metabolism dependent) to reactive metabolites has a role to play in the pathogenesis of serious ADRs, but whether it is the predominant mechanism or one that plays an accessory role will depend on the drug, host, and environmental factors.

### The pharmacologic interaction hypothesis of drug hypersensitivity

Pichler^74^ has proposed an alternative hypothesis based on direct binding of the parent drug to the T-cell receptor or the HLA molecule. This is termed the pharmacologic interaction (p-i) hypothesis and requires the drug to bind to these receptors with high affinity but in the absence of innate immune activation, metabolism, or antigen processing.^75^ Indeed, it has been proposed that HLA restriction, as has been demonstrated with many drugs (outlined above), is likely to occur only when a drug is able to bind to one HLA allele but not when the drug is converted to a reactive metabolite because this will generate a large number of antigenic determinants.^75^ However, this ignores the increasing number of HLA alleles that have been associated with drugs causing liver injury in which metabolism might play an important role (Fig 1).

Interaction with T-cell receptors, which results in T-cell activation (but not antibody formation), has been shown with a number of drugs, including sulfamethoxazole, abacavir, carbamazepine, lamotrigine, lidocaine, and allopurinol.^76^ However, the evidence is largely based on *in vitro* experiments, and although direct T-cell interaction is likely to occur *in vivo* as well, it does not fully simulate the complex situation seen *in vivo* in which drug concentrations will vary in different tissues and there will be differing amounts of metabolites present in different subjects, which will include variability in the formation of drug-protein conjugates. Thus there is a need to rationalize how the 2 potential mechanisms might operate together under different circumstances. This can be done by considering the drug sulfamethoxazole, an antimicrobial used for the treatment of urinary tract infections and *Pneumocystis jirovecii* pneumonia.

Sulfamethoxazole has been used to define the chemical basis of drug (metabolite) hypersensitivity reactions because its reactive metabolite, nitroso sulfamethoxazole, can be synthesized^77^ and integrated into functional *in vitro* T-cell assays at nontoxic concentrations.^78^ *In vivo* cytochrome P450–mediated metabolism of sulfamethoxazole generates an unstable but not protein-reactive hydroxylamine metabolite that can be detected in plasma.^79^ The hydroxylamine undergoes rapid spontaneous oxidation to nitroso sulfamethoxazole, an electrophile that can bind covalently to cysteine residues in both extracellular and cellular proteins.^80-82^ Formation of adducts can induce apoptotic and necrotic cell death and the release of mediators that promote dendritic cell maturation.^83-85^ The binding of nitroso sulfamethoxazole to protein produces multiple adduct structures and therefore a multitude of potential antigens. Whether this is a potential explanation for the lack of an HLA association with sulfamethoxazole hypersensitivity is unclear.^86^

Nitroso sulfamethoxazole is chemically unstable in cell culture medium; its half-life is less than 30 minutes,^77,78^ and therefore cellular exposure to nitroso sulfamethoxazole in T-cell assays is likely to be 10- to 50,000-fold lower than the parent compound. Despite this, nitroso sulfamethoxazole–responsive T cells have been detected in the periphery and skin of 100% of patients with sulfamethoxazole hypersensitivity.^87-90^ In patients with milder skin reactions (eg, maculopapular exanthema), CD4^+^ T cells are preferentially activated, whereas CD8^+^ T cells are more readily detected in patients with SJS/TEN. T cells from hypersensitive patients are also activated with the parent drug sulfamethoxazole through direct interaction with MHC molecules, specific T-cell receptors, or both.^91^ Some of these T cells were found to be highly structurally specific in that different sulfonamide derivatives can bind to the T-cell receptor, but only sulfamethoxazole results in activation. This has been attributed to its orientation being similar to that of the hapten formed with nitroso sulfamethoxazole.^92^

After *in vitro* priming of naive T cells with nitroso sulfamethoxazole, it is possible to prime naive CD4^+^ T cells from 100% of healthy human donors in 7 days if (1) the drug-derived antigen was presented by dendritic cells, (2) regulatory T cells were removed from the assay, and (3) costimulatory signals were provided through the use of LPS/TNF-α.^93^ Furthermore, the magnitude of the T-cell response could be enhanced dramatically through blockade of programmed cell death 1/programmed cell death ligand 1 signlling.^94^ Feeding regulatory T cells back into the system effectively blocked nitroso sulfamethoxazole–specific T-cell priming. Hence levels of costimulatory/coinhibitory signaling at the time of drug exposure might be another important determinant of whether a pathogenic T-cell response develops.

Taken together, these observations show that T cells circulating in the same hypersensitive patients are activated by different chemical entities through different pathways (both stimulatory and inhibitory), which is consistent with drug- and metabolite-specific T cells being part of the spectrum of a polyclonal T-cell response. Therefore it is impossible to identify the drug-derived antigen that actually initiated the initial T-cell response in a patient from retrospective analysis of the frequency of memory T cells (recall). Furthermore, at present, we cannot be certain which particular T cells are the actual effector cells during a hypersensitivity reaction *in vivo* when the target organ is usually the skin or, less commonly, the liver.

### Methods to characterize drug/HLA interactions

Drugs that show HLA associations can be tested for HLA binding by using *in vitro* assays. A cell-free system with purified HLA molecules can be used to measure drug effects on peptide-binding affinity by using a positional scanning peptide library approach.^95^ In this approach binding assays are established in which HLA molecules are incubated with drug and peptide mixtures in which a peptide position is fixed to a single amino acid and randomly at all other positions. For 9-mer peptides (20 amino acids × 9 positions), 180 peptide mixtures can be used in HLA-binding assays in the presence and absence of drug. For example, peptide libraries in which position 9 (P9) was fixed to be V or I (and random at all other positions) were found to bind HLA-B*57:01 with higher affinities in the presence of abacavir compared with the absence of the drug.^95^

Elution of HLA-bound peptides from drug-treated cells is an informative method to characterize drug/HLA interactions. In this method cells expressing the HLA molecule of interest are grown to large numbers (>10^9^) when treated with either the drug or vehicle control. The HLA molecules are immunopurified, and bound peptides are subsequently eluted and then characterized by means of mass spectrometry and sequencing. Comparison of peptides eluted from HLA in the presence and absence of drug can be used to measure the effects of the drug on the HLA-bound peptide repertoire. For example, HLA-B*57:01 typically exhibits a preference for 9-mer peptides with bulky aromatic side chains (such as W or F) at P9. However, peptides eluted from HLA-B*57:01 after isolation from abacavir-treated cells exhibit an altered binding preference: nonamer peptides with short aliphatic side chains at P9 (I or V). These data show that abacavir treatment of cells results in alteration of the repertoires of HLA-B*57:01–bound peptides.^95,96^ Alteration of the repertoire of HLA-bound peptides might be due to direct (eg, by drug/HLA binding) or indirect (eg, by altering protein expression, metabolism, or both) effects. The peptide elution method can be used to measure the effects of a drug on the HLA-bound peptide repertoire, regardless of whether the effect is direct or indirect. These aspects in relation to abacavir, together with the concepts of heterologous immunity, alloreactivity, and polyspecificity, are covered by White et al^34^ in this issue of the *Journal* and will not be repeated here.

## Conclusions

The discovery of MHC restriction in drug hypersensitivity has provided an opportunity to delve further into the fundamental chemical basis of how a drug might initiate such events in a patient and raise broader pharmacologic questions with regard to the disposition and effector functions of drug-related antigens. Proteins are processed by heterogeneous intracellular pathways, leading to the generation of peptide–MHC class I complexes and peptide–MHC class II complexes. For both pathways, the peptides are mostly buried in the peptide-binding groove, and the peptide is an integral part of the folded protein complex, part of which is recognized by T-cell receptors. Endogenous peptides can be altered posttranslationally through a variety of chemical processes, some of which might be disease induced,^97,98^ and thus contribute to allergy or autoimmune disease. The hapten hypothesis would suggest that a drug (metabolite) covalently modifies a protein before processing with presentation of drug peptide. Although drug-hapten protein complexes have now been detected *in vivo* for a number of drugs, characterization of drug peptides in the context of MHC binding and T-cell activation has not been defined. The p-i hypothesis provides an alternative mechanism that has been demonstrated to operate *in vitro*, but there is no direct experimental evidence that it operates *in vivo*. More recently, studies with abacavir have shown that it might occupy a space below peptides expressed by particular HLA alleles, thus leading to altered peptide presentation and autoimmunity. The difference between the altered peptide/p-i hypotheses and the hapten hypothesis is that the former do not depend on covalent interaction *in vitro*.

**Fig 3:**
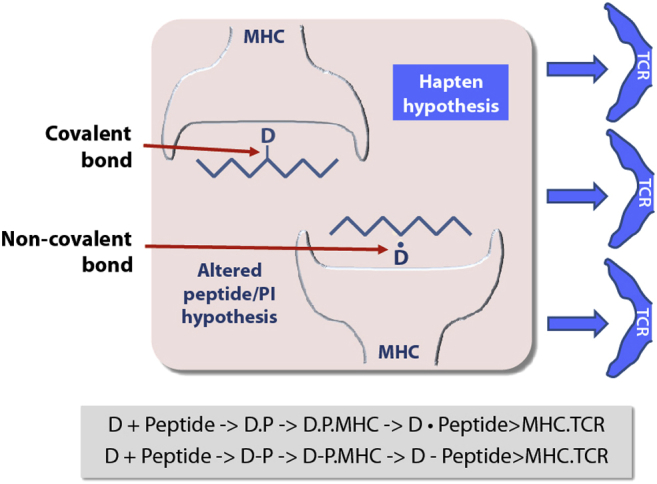
A simple scheme to illustrate how a ternary complex between the drug, MHC molecule, and peptide can be assembled to stimulate a T-cell response. The interaction between the peptide and drug can be either covalent (D-) or noncovalent (D.). It is possible that in the *in vivo* situation both models could be occurring simultaneously, but we do not have the precise chemical tools to determine this.

Although it is important to identify the mechanisms of drug hypersensitivity, perhaps too much emphasis is being placed on the differences between the various hypotheses rather than focusing on the fact that the mechanisms are complementary and might at least partly account for the heterogeneity of the immune response seen in different patients. For example, even with abacavir, although there is evidence of peptide-binding displacement^95^ and alloimmunity,^99^ abacavir protein haptens have also been detected *in vivo*^100^; the role of these different processes *in vivo* in patients with abacavir hypersensitivity is thus unclear, and it is of course possible that all 3 are important. Thus what is being suggested (Fig 3) is that an assembly of the MHC molecule, peptide, and drug leads to a complex maintained by a variety of chemical bonds (which could include covalent, coordinate, hydrogen, and hydrophobic bonds). The thermodynamic stability of the complex will be determined by the totality of the binding energies and not the type of bond *per se*. Furthermore, it seems highly likely that the epitopes recognized by various T cells will have variable composition of substructures in the drug, peptide, and MHC molecule, some of which might not contain the drug at all.

Finally, we know little or nothing about the nature of the MHC-drug-peptide complex that elicits target organ toxicity in the afferent arm of the immune response *in vivo*. We need to know more about the relationship between total drug disposition and the constitution of drug-peptide-MHC complexes in both the afferent and efferent arms of the immune response to define thresholds (drug, metabolite, and drug-protein conjugate) in early drug discovery that can be reasonably expected not to pose a risk in the clinic.
